# Rheumatoid Factor, Complement, and Mixed Cryoglobulinemia

**DOI:** 10.1155/2012/439018

**Published:** 2012-08-26

**Authors:** Peter D. Gorevic

**Affiliations:** Mount Sinai School of Medicine, Division of Rheumatalogy, Annenberg Building, Room 21-056, One Gustave L. Levy Place, New York, NY 10029-6574, USA

## Abstract

Low serum level of complement component 4 (C4) that occurs in mixed cryoglobulinemia (MC) may be due to in vivo or ex vivo activation of complement by the classical pathway. Potential activators include monoclonal IgM rheumatoid factor (RF), IgG antibodies, and the complexing of the two in the cold, perhaps modulated by the rheology and stoichiometry of cryocomplexes in specific microcirculations. There is also the potential for activation of complement by the alternative and lectin pathways, particularly in the setting of chronic infection and immune stimulation caused by hepatitis C virus (HCV). Engagement of C1q and interaction with specific cell surface receptors serve to localize immune complexes (ICs) to the sites of pathology, notably the cutaneous and glomerular microcirculations. Defective or saturated clearance of ICs by CR1and/or Fc receptors may explain persistence in the circulation. The phlogistic potential of cryoprecipitable ICs depends upon the cleavage of complement components to generate fragments with anaphylatoxin or leukocyte mobilizing activity, and the assembly of the membrane attack complex (C5b-9) on cell surfaces. A research agenda would include further characterization of the effector arm of complement activation in MC, and elucidation of activation mechanisms due to virus and viral antigens in HCV infection.

## 1. Introduction

**Figure 1:**
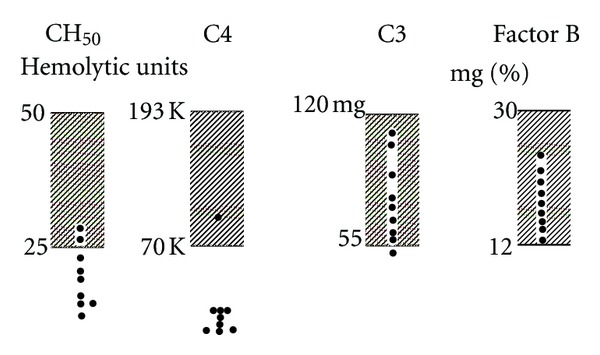
C3, C4, and factor Bb levels determined by hemolytic assay in patients with type 2 MC (4).

Mixed cryoglobulins (MCs) are cold-precipitable rheumatoid factors (RFs) that are easily identifiable and characterized by immunofixation of cryoprecipitate obtained from serum carefully collected from blood kept at and allowed to clot at core body temperature, and then cooled to 4°C [1]. Type 2 MCs are almost invariably composed of monoclonal IgM kappa RF and polyclonal IgG, and it is the complexing of the two that is a requisite for the formation of cold-precipitable immune complexes (ICs); both the IgM heavy-and light-chain variable regions display a striking clonality that is mirrored in cross reactive idiotypes (CRIs) as well as mu heavy chain and kappa light-chain V-region gene usage. Type 2 MCs are heavily represented among cryoglobulins associated with chronic hepatitis C virus (HCV) infection, and those found in patients with primary Sjögrens syndrome, both of which may be complicated by clonal B-cell proliferations and specific types (e.g., mucosa-associated (MALT); Splenic) of non-Hodgkin's lymphoma [2]. Among patients with type 2 MCs associated with HCV, prominent associations with extrahepatic disease manifestations such as leukocytoclastic vasculitis, arthropathy, neuropathy, and membranoproliferative glomerulonephritis have been found in multiple series. Complement abnormalities were described in early series of “essential mixed cryoglobulinemia” [3], and type 2 MCs are likely to have a striking complement profile notable for normal or low levels of component 3 (C3) and often undetectable levels of component 4 (C4); the latter (Figure 1) provides a “signature” which may in fact be used to anticipate the presence of significant (>1 mg/mL) amounts of type 2 cryoglobulin in blood [4]. The purpose of this paper is to update older information with regard to complement measurements in type 2 MC, with particular attention to the various effects of HCV infection and the central role of RF.

## 2. The Complement System

The complement system comprises 30 serum and cell-surface proteins tightly regulated to respond to activators by three independent pathways (classical: CP, alternative, AP, and Mannan-binding lectin: MBL), evolved primarily to recognize and destroy pathogenic microorganisms [5]. Temperature-dependent activation of both CP and AP in vitro has been reported among mixed (IgM-IgG, IgM-lipoprotein) and monoclonal (IgG) cryoglobulins. Activation of AP has been correlated with the presence of IgA in mixed cryoglobulins and with an IgG3 monoclonal cryoglobulin occurring in a patient with membranoproliferative glomerulonephritis [6]. The selective depression of C4 noted in type 2 MC implicates the CP and is reflected in an extended serum profile, which includes variably low levels of C1q and C2, normal levels of factor Bb (Factor 1), and elevated levels of MBL; C3 levels may be normal, except in patients with severe disease manifestations (glomerulonephritis, neuropathy) [7]. In HCV-associated MC, this profile (a) correlates inexactly with the level of cryoglobulin and titer of RF, (b) may occasionally be found in the absence of a detectable cryoglobulin, (c) may occur in the absence of RF in the serum supernatant after cryoprecipitation, (d) correlates only poorly with symptomatology in serial studies, and (e) may persist with cryoglobulinemia after apparent clearance of the virus [8–10]; these observations suggest a complexity of pathways to C4 depletion extending beyond IC activation and HCV infection. The mechanism responsible for the selective depression of C4 remains unclear; whereas a novel control mechanism involving Cb-binding protein (C4-bp) and C3b inactivator was implicated in one early study [11], this was not reflected in the levels of C4-bp in sera of patients with MC [12]. Whether cryo-RF might interfere with complement activation at the level of C3 has not been addressed.

## 3. Rheumatoid Factors 

Rheumatoid factors are IgM antibodies with specificity largely for the Fc portion of IgG; potential triggers to mRF production include (a) direct infection by virus, (b) chronic antigenic stimulation by ICs, (c) stimulation in the form of repetitively arranged epitopes on viral particles, or (d) molecular mimicry. Early studies suggested additional reactivities of MC IgM with idiotypic determinants in the F(ab')2 of MC IgG, possibly reflecting the fact that in MC the IgG is reactive with viral antigens [13], some of which can also be demonstrated within the cryoprecipitates [6]. ICs containing IgG and IgM isotypes are well known to be activators of the CP, providing several mechanisms by which C1q binding to the CH_2_ domain of IgG, and/or to the CH_3_ or CH_4_ domains of IgM may occur in MC [5]. RFs in type 2 MC are distinct from those found in rheumatoid arthritis (RA) with regard to virtually universal cold perceptibility, clonality (CRIs and skewing of V-region gene usage), and in that the antiglobulin activity is unique to the IgM isotype [14]. Direct activation of complement by locally produced RF-containing ICs has been well demonstrated in the joint space of patients with RA, resulting in depression of C4 [15], but has not been found in serum, except for a small number of patients with very severe (and likely overlap) disease, characterized by recurrent infections [16]. By contrast, serum levels of C4, and more specifically C3, may be elevated in serum of patients with RA by virtue of their being acute-phase reactants (APRs), a phenomenon that has also been described in HCV infection, and used to monitor efficacy of treatment [17]; elevation of complement component APRs might mask more subtle activation due to ICs in the circulation unless specific cleavage products (e.g, C3b and C5a) are also assayed.

**Figure 2:**
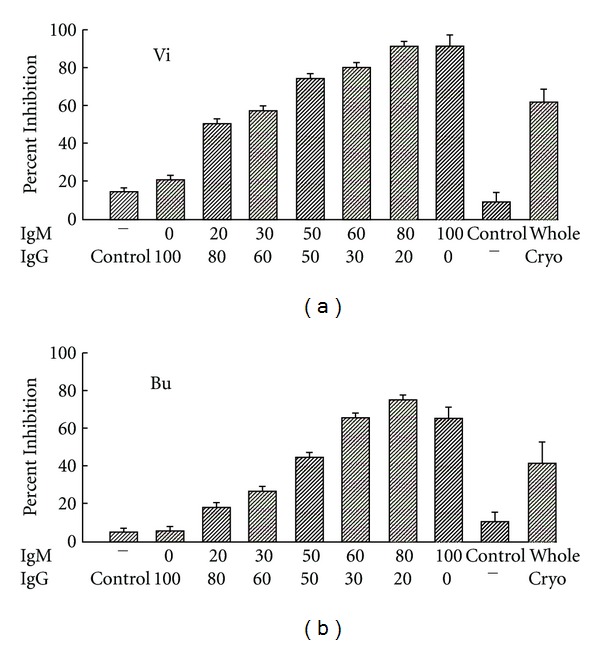
Total hemolytic complement activity was determined after reconstitution of varied molar ratios of separated IgM and IgG from two type 2 cryoglobulins (Vi; Bu). Separated IgM was 100% monoclonal IgMk with RF activity; the IgG fraction had immunoblot reactivity to multiple HCV linear antigens. Isolated cryoglobulin was significantly (40 and 60% total serum RNA by RT-PCR) enriched in HCV RNA. Separated IgM and IgG fractions were mixed at 37 and cooled O/N to 4 C. Results are compared to control IgG and IgM, the latter from a patient with Waldenströms macroglobulinemia without RF activity. Both cryoglobulins were obtained from patients chronically infected with HCV, with active cutaneous vasculitis and membranoproliferative glomerulonephritis. (courtesy of Dr. B. Ghebrehiwet).

MC formation provides a fertile substrate for in vitro and, by extension, potential for in vivo, complement activation. Both IgG and IgM may be recognized by the globular heads of C1q, which has been identified as a constituent of cryoprecipitates in some studies. Although binding of C1q to monomeric IgG in cryocomplexes might be anticipated to be offset by more effective binding to IgM, this could be mitigated by clustering of IgG in the ICs, complex formation of IgG with viral antigens, and increased representation of the IgG3 subclass, which is known to be most effective for CP activation [5, 6]. An additional factor might be the stoichiometry of IgG and IgMRF in the ICs, which has been reported to influence in vitro cryocomplex formation. Among IgM-IgG mixed cryoglobulins, fractionation experiments have established that the majority of anticomplementary activity is associated with IgM RF; in mixing experiments of dissociated and purified IgG and IgM from two patients with HCV-associated type 2 MC, the bulk of complement activation appeared to localize to the IgMRF constituent (Figure 2). Nevertheless, in vitro studies indicated that IgM RF/IgG immune complexes may not fix C3 and C4 efficiently in spite of fluid-phase activation [18], and appear to bind poorly to erythrocyte complement receptor 1 (CR1; CD35), the most abundant C3b/C4b receptor in blood [19]. A mechanism for activation due to MBL-MASP2 binding to underglycosylated RF, or direct activation by HCV RNA, has not been formally tested. Since the valence of IgM is five, in spite of the potential for 10 antigen binding sites, it seems possible that the stoichiometry of cryocomplexes might be influenced by the binding affinity for determinants on the IgG, the aggregation state of IgG, specificity for other components of the cryocomplex, cold-related aggregation, and the rheology of specific microcirculations such as exists in the skin and the afferent arterioles of the glomeruli, where increased protein concentration and skin temperatures significantly below core levels might facilitate the occurrence of such molecular events and cell interactions, thus allowing more robust complement activation at the sites of pathology [20].

## 4. Cold-Dependent Activation of Complement**** (CDAC)

In sera manifesting CDAC, a similar profile of low CH_50_ and hemolytic C4 with normal hemolytic C5-C9 is seen at 4°C, with normal values being obtained in EDTA-treated plasma and in serum kept at 37°C. First described as an in vitro phenomena in occasional sera [21], it was subsequently shown to be prominently associated with HCV infection, and not with cold-associated symptomatology (raynaud or acrocyanosis). It does, however, correlate with the degree of liver damage and inversely with a response to treatment with interferon-alpha. In contradistinction to cryoglobulinemia, C1q and C4 antigenic levels are normal in CDAC. Cryoprecipitates (cryoglobulins or cryofibrinogens) are usually not found. CDAC may be consequent to HCV-antibody-mRF complexes with differing stoichiometry [22, 23]. CDAC, RF, cryoglobulinemia, and elevated levels of IgM-containing ICs are all prevalent in HCV infection. Dissociation of the thermal properties of cryoprecipitation and complement activation has been noted in specific instances in which it has been studied.

## 5. Constituents of Mixed Cryoglobulins

Early studies of mixed cryoglobulins associated with severe RA provided the first indication that as much as one quarter to one-third of the cryoprecipitable material was nonimmunoglobulin [24]. In HCV-associated MC, other constituents include C1q, C-reactive protein (CRP), HCV antigens, and molecules of the lectin complement pathway (MBL and MBL-associated serine protease-1), the latter associated with membranoproliferative glomerulonephritis [7, 25]. Though the quantitative contribution of these other components has not been determined, they provide alternative routes for activation of complement via the CP or MBL, for example, activation of C1q by CRP in the presence of phosphocholine produced by apoptotic cells. Although most of the demonstrable reactivity to HCV antigens in MC appears to reside in the IgG fraction [26], the possibility remains that significant antibody activity—and by extension anticomplementary activity—to conformational antigens (i.e, envelope proteins; encapsulated or unencapsulated virus) might be retained in the IgM RF fraction [27].

## 6. Phlogistic Potential of Mixed Cryoglobulins

The ability of cryoaggregates to generate vasoactive substances and proinflammatory mediators to produce tissue lesions is suggested by elevated levels of complement fragments with anaphylatoxin activity (C3a, C5a) in serum, as well as the ability of isolated cryoproteins to activate basophils, cause platelet aggregation, and interact with kallikrein-kinin in vitro [6]. In addition, C5a is a potent chemotactic factor that might be responsible for the influx of inflammatory cells to the site of complement activation. Direct activation of C1 by kallikrein provides an independent mechanism for activation of both the CP and AP separate from a role for ICs in MC [5].

## 7. Defective Clearance of Cryoglobulins

 C1q binding has been used as an assay for the identification of both cryoprecipitable and noncryoprecipitable ICs in the sera of patients with MC [28]. Defective clearance of cryoglobulinemic ICs by Fc receptor-mediated mechanisms correlates with persistence in the circulation, and the clinical manifestations of nephritis and neuropathy [4]; cryoglobulin-induced cytokine production via Fc gamma IIa ligation in MC has been proposed as a mechanism for the generation of TNF-alpha and IL-10, and for the growth of malignant B-cells in this disorder [29]. Similarly, decreased erythrocyte CR1/CD35 (receptor for C3b and membrane cofactor protein) in both MC and chronic liver disease has been linked to deficient reticuloendothelial system (RES) clearance, defective cleavage of C3b and C4b, and discordant complement activation at different concentrations and temperatures of RF [19, 30]. CR1 numbers were decreased when peripheral blood erythrocytes were analyzed with a monoclonal antibody to this receptor. Defective immune adherence and elimination of other ICs, such as hepatitis B surface antigen/antibody, may lead to trapping in tissues, resulting in clinical vasculitis or nephritis. Inhibition of neutrophil and monocyte chemotaxis, as well as bactericidal function, has been attributed directly to the effects on these cells of cryoprotein constituents and may relate to an increased incidence of infections in some patients [31].

## 8. Hepatitis C Virus and Complement

The detection of HCV core antigen in cryoprecipitates has been linked to the presence of unencapsulated nucleocapsid particles as a constituent of MCs [27]. A receptor for the globular head of the C1q molecule (gC1q-R) appears to be a natural ligand for HCV core antigen [32]. The structural similarity between HCV core antigen and C1q may explain the presence of cross-reactive anti-C1q in HCV-associated MC [33]. MCs provide a platform for the formation of qC1q-R-HCV core complexes, and their localization to sites of vasculopathy, where they might bind to receptors on endothelial cells, as demonstrated by immunohistology [34]. Anti-C1q and anti-CRP antibodies have been linked to disease severity, and to autoimmunity in HCV infection [33, 35]. In addition to CP activation, the binding of MBL to HCV envelope glycoproteins E1 and E2 [36] might lead to activation of the lectin pathway due to homology of MBL to C1q, resulting in cleavage of C4 and C2 following interactions with MBL-associated proteases [5]. This has been corroborated in immunohistological studies of HCV-associated membranoproliferative glomerulonephritis [7]. An alternative mechanism for the depression of C4 and perhaps C3 has been provided by the demonstration of the ability of HCV viral proteins to regulate synthesis of C3 and C4 via specific transcriptional repression [37, 38].

## 9. Membrane Attack Complex (MAC)

The MAC is dependent on the cleavage of C5 into C5a and C5b leading to the assembly of C6–9 and lytic activity targeting membranes at the site of tissue pathology. Relatively little information has been accumulated to implicate terminal complement activation and the generation of MAC in tissue lesions associated with extrahepatic HCV infection.

## 10. Discussion

The low level of C4 characteristic of some sera from patients with type 2 MC may be due to activation and cleavage, clearance abnormalities, or reduced synthesis. Activation may proceed via the CP, AP, or MBL pathways, masked by the increased synthesis of this APR due to the inflammation of liver damage and/or IC disease. Activation may be consequent to the IgM RF and/or IgG fractions of type 2 MC, complex formation, and other constituents of cryoprecipitates, including HCV viral antigens, viral RNA, and CRP. Cryoglobulin formation provides a marker for the persistence of ICs in the circulation of affected individuals, as well as for the development of occlusive and/or inflammatory vasculopathy, particularly in the skin. Persistence of ICs may result from defective or saturated clearance mechanisms involving complement (CR1 and other), immunoglobulin Fc (RIIa and other), and/or C1q (gC1qR and other) receptors. Tissue damage due to complement activation requires the generation of fragments with anaphylatoxin (C3a, C4a, and C5a) and leukocytosis mobilizing (C3d and C3e) activity, and the engagement of their specific receptors; it is reflected in footprints for the assembly of the MAC complex at sites of tissue injury. Lastly, polymorphisms of proteins that tightly regulate the complement system might be interrogated to determine the environmental and genetic determinants of complement abnormalities characteristic for type 2 MC [39, 40].
